# A murine model of Charcot-Marie-Tooth disease 4F reveals a role for the C-terminus of periaxin in the formation and stabilization of Cajal bands

**DOI:** 10.12688/wellcomeopenres.13673.1

**Published:** 2018-03-01

**Authors:** Diane L. Sherman, Peter J. Brophy

**Affiliations:** 1 Centre for Discovery Brain Sciences, University of Edinburgh, Edinburgh, EH16 4SB, UK

**Keywords:** peripheral nerve, myelination, Charcot-Marie-Tooth Disease, periaxin, Cajal bands

## Abstract

Charcot-Marie-Tooth (CMT) disease comprises up to 80 monogenic inherited neuropathies of the peripheral nervous system (PNS) that collectively result in demyelination and axon degeneration. The majority of CMT disease is primarily either dysmyelinating or demyelinating in which mutations affect the ability of Schwann cells to either assemble or stabilize peripheral nerve myelin. CMT4F is a recessive demyelinating form of the disease caused by mutations in the *Periaxin* ( *PRX*) gene *.* Periaxin (Prx) interacts with Dystrophin Related Protein 2 (Drp2) in an adhesion complex with the laminin receptor Dystroglycan (Dag). In mice the Prx/Drp2/Dag complex assembles adhesive domains at the interface between the abaxonal surface of the myelin sheath and the cytoplasmic surface of the Schwann cell plasma membrane. Assembly of these appositions causes the formation of cytoplasmic channels called Cajal bands beneath the surface of the Schwann cell plasma membrane. Loss of either Periaxin or Drp2 disrupts the appositions and causes CMT in both mouse and man. In a mouse model of CMT4F, complete loss of Periaxin first prevents normal Schwann cell elongation resulting in abnormally short internodal distances which can reduce nerve conduction velocity, and subsequently precipitates demyelination. Distinct functional domains responsible for Periaxin homodimerization and interaction with Drp2 to form the Prx/Drp2/Dag complex have been identified at the N-terminus of Periaxin. However, CMT4F can also be caused by a mutation that results in the truncation of Periaxin at the extreme C-terminus with the loss of 391 amino acids. By modelling this in mice, we show that loss of the C-terminus of Periaxin results in a surprising reduction in Drp2. This would be predicted to cause the observed instability of both appositions and myelin, and contribute significantly to the clinical phenotype in CMT4F.

## Introduction

Charcot-Marie-Tooth (CMT) disease is a group of genetically heterogenous neuropathies that comprise the commonest disorders of the peripheral nervous system (PNS) ^1^. CMT4F is a demyelinating autosomal recessive type caused by about 24 different mutations in the *Periaxin* ( *PRX*) gene that manifests with motor and sensory disturbances ^2– 13^. Periaxin forms a complex with Dystrophin-Related Protein 2 (Drp2) and Dystroglycan (Dag) and this Prx/Drp2/Dag complex assembles adhesive appositions between the abaxonal surface of PNS myelin and the Schwann cell plasma membrane that are surrounded by cytoplasm-filled Cajal bands ^14, 15^. The appositions and Cajal bands are lost either in the complete absence of Periaxin or Drp2, or if the interaction between these two proteins is prevented, and this results in disruption of the myelin sheath ^14– 16^. By comparison with Drp2, loss of Periaxin leads to a more severe phenotype in mice, involving more extensive demyelination and reductions in internodal length associated with reduced nerve conduction velocities, all of which are consistent with the distinct clinical severities observed in man ^14, 15, 17, 18^.

An intriguing aspect of the *Prx* gene is that more than 90% of the protein is encoded by its terminal exon ^19^. Since most CMT4F nonsense or frame-shift mutations are located in this exon, the corresponding mRNAs that encode premature stop codons are likely to escape nonsense-mediated RNA decay and result in the production of a mutant version of the Periaxin protein ^20^. Proof that this is the case has been obtained using sural nerve biopsies from two families harbouring distinct mutations ^8, 9^. One of these mutations has been shown to result in the production of C-terminally truncated Periaxin lacking the last 391 amino acids ^4, 7, 9, 21^. Since the N-terminus of Periaxin has been primarily implicated in the assembly of the Prx/Drp2/Dag complex thus far ^15, 16, 18^, we have explored whether loss of the C-terminus might also contribute to the formation and/or stabilization of the membrane appositions responsible for the construction of Cajal bands.

## Methods

### Mice

All animal work conformed to United Kingdom legislation (Scientific Procedures) Act 1986, and to the University of Edinburgh Ethical Review Committee policy. The production of transgenic mice, expressing the cDNA encoding full-length mouse Periaxin ( *PrxTg*) under the control of a rat Mpz promoter and generated by RT-PCR with a FLAG tag fused at its 5´ end, has been described previously ^15^. A transgenic line expressing a FLAG-tagged, truncated mouse Periaxin lacking the C-terminal 391 amino acids was generated in similar fashion ( *ΔCPrxTg*). The presence of the N-terminal FLAG tag allowed for the unambiguous identification of transgenic proteins. After backcrossing to C57BL/6 (Harlan) for at least six generations, these mice were interbred with *Prx ^-/-^* mice so that the transgenes were expressed on a Periaxin-null background as previously described ^15^. Males and females were used for all experiments and the age and number of the animals are described in the figure legends. For Western blotting and immunocytochemistry animals were sacrificed humanely by a schedule 1 method (cervical dislocation followed by exsanguination) in compliance with the UK Animal (Scientific Procedures) Act, 1986. For electron microscopy mice were anaesthetized with halothane (as approved for PPL P0F4A25E9 under the UK Animal (Scientific Procedures) Act, 1986) and perfused with fixative as described ^15^. All mice were housed in individual ventilated cages to ensure optimal health status and the health status of sentinel mice was routinely screened every six months.

### Western blotting, immunocytochemistry, histology and electrophysiology

Western blotting of peripheral nerve lysates prepared from sacrificed mice was using rabbit polyclonal antibodies directed at the N-terminus (1:20,000) or sheep polyclonal antibodies (1:10,000) to the C-terminus of mouse Periaxin (5 μg lysate protein) and rabbit anti-Drp2 polyclonal antibodies (1:3000) (15 μg lysate protein) ^16, 19^. Rabbit polyclonal antibodies versus γ-Actin used for Western Blotting (1:10,000) were raised against a peptide comprising the N-terminus of γ-Actin with an additional C-terminal cysteine (EEEIAALVIDNGSGC) coupled to Keyhole Limpet hemocyanin as described ^19^. Immunostaining of teased peripheral nerve fibres with rabbit anti-Drp2 polyclonal antibodies (1:200) ^16^, and light microscopy of toluidine blue-stained transverse sections of nerve were all performed using quadriceps nerves as previously described, and abnormal profiles were evaluated by examining a minimum of 491 myelinated axons per animal ^15, 18^. Internodal lengths (a minimum of 96 per animal) and nerve conduction velocities (a minimum of 2 per animal) were measured as described previously ^14^.

### Cross-linking, immune precipitation of periaxin and mass spectrometry

Formaldehyde crosslinking prior to immuneprecipitation has been described ^22^. Freshly prepared 4% paraformaldehyde in 100 mM Sorenson’s phosphate buffer pH 7.4 was prepared and frozen in aliquots. Sciatic and quadriceps nerves from each mouse were desheathed and teased in PBS containing phosphatase inhibitors. After removal of the PBS, a 0.5% formaldehyde solution in 100 mM phosphate buffer (1 ml) was added and the nerves were incubated for 9 min with agitation at room temperature. After sedimentation by centrifugation for 1 min, 0.5 ml of 1.5 M glycine was added to the pellet. Nerves were further washed at 4°C with 1.25 M glycine and PBS, followed by a final wash with PBS. Nerves were then lysed in 50 μl of 2% SDS and incubated at 65°C for 10 min. Lysates were collected and stored at -80°C.

Periaxin was immuneprecipitated from the nerves of 2 mice (100 μl) after first warming lysates to 55°C for 2 min followed by dilution with 400 μl of Solution A as previously described ^23^. The diluted lysate was incubated overnight with washed FLAG agarose beads (20 μl) (Sigma A2220) at 4°C with rotation. The beads were extensively washed with Solution A without Triton X-100, resuspended with 30 μl NuPage sample buffer containing DTT (Invitrogen), and warmed at 65°C for 10 min. The sample was briefly electrophoresed in NuPage gel and after staining with SimplyBlue SafeStain (Invitrogen) according to the manufacturer’s instructions, the high molecular weight cross-linked bands were excised and processed for mass spectrometry.

Three separate samples were analysed for both *PrxTg/Prx ^-/-^* and *ΔCPrxTg/Prx* ^-/-^ mice (a total of 6 mice per genotype). After tryptic digestion of the gel bands, peptide extracts were dried, resuspended in MS-loading buffer (0.5% trifluoroacetic acid in water) and filtered using Millex filters before HPLC-MS analysis. Analysis was performed using an online system of a nano-HPLC (Dionex Ultimate 3000 RSLC, Thermo-Fisher) coupled to a QExactive mass spectrometer (Thermo-Fisher) with a 300µm × 5 mm pre-column (Acclaim Pepmap, 5µm particle size) joined with a 75µm × 50cm column (Acclaim Pepmap, 3µm particle size). Samples were run using a 90 min gradient and data from MS/MS spectra was searched using MASCOT against a Uniprot Mouse database. Progenesis (Nonlinear Dynamics) was used for label-free quantitation. Search results were exported using a significance threshold (p-value) of less than 0.05 and a peptide score cut off of 20. All samples were normalized for protein content.

### Statistical analysis

Prism 6 (GraphPad Software, version 6.0g) was used to evaluate statistical significance using an unpaired t-test with Welch’s correction and to generate all graphs. 

## Results

**Figure 1.**
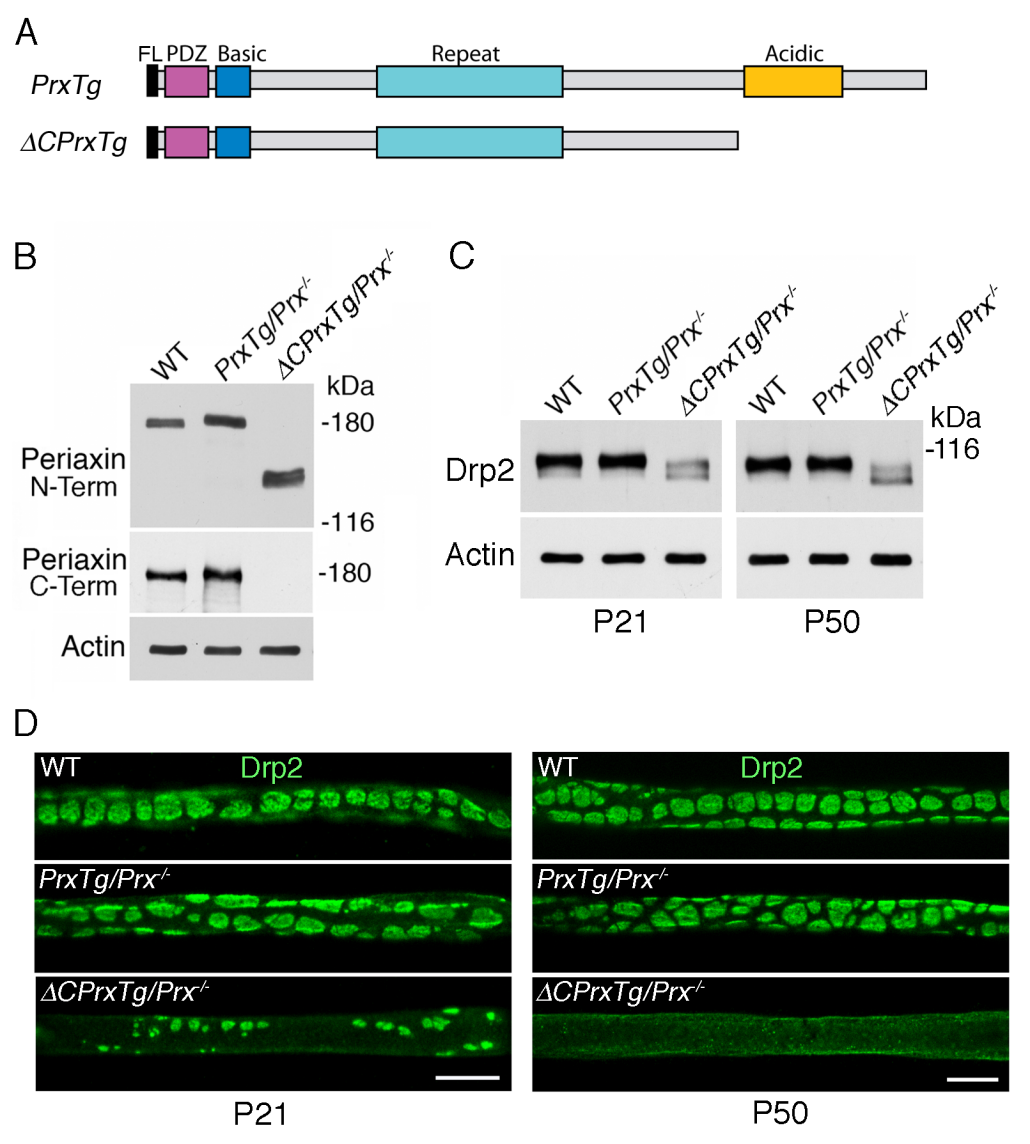
C-terminal truncation of Periaxin disrupts appositions between the abaxonal surface of the myelin sheath and the Schwann cell plasma membrane formed by the Prx/Drp2/Dag complex. ( **A**) Diagram showing the proteins encoded by the transgenes *PrxTg/Prx ^-/-^* and *ΔCPrxTg/Prx* ^-/-^ respectively. The locations of the FLAG tag (FL), and PDZ, basic, repeat and acidic domains are also shown, of which the PDZ and basic domains are known to be essential for apposition assembly ^16, 18, 19, 23^. ( **B**) Western blotting with an antibody directed to the N-terminus of Periaxin detects the wild-type (WT) protein and both transgenic proteins when expressed on a Periaxin-null background, whereas only the full length WT and transgenic protein was detected by an antibody against the C-terminus of Periaxin. Peripheral nerve lysates were from P21 animals. γ-Actin is the loading control. ( **C**) Western blots showing decreases in the level and extensive dephosphorylation (lower band) of Drp2 in *ΔCPrxTg/Prx* ^-/-^ mice *.* The extent of Drp2 dephosphorylation increased from P21 to P50. ( **D**) Drp2-positive appositions were sparse but detectable by immunofluorescence at P21 but by P50 they were gone, and this coincides with the greater degree of Drp2 dephosphorylation at this later age (see **C**). Size marker = 10 μm.

Proteins with predicted sizes were expressed from the full length and truncated *Prx* transgenes in a Periaxin-null background ( Figures 1A and 1B). Both proteins were detected by Western blotting using an antibody directed to the N-terminus, whereas only the full length protein was detected by an antibody against the C-terminus of the wild-type protein ( Figure 1B). Although the levels of Periaxin’s binding partner Drp2 were normal in the quadriceps nerves of control *PrxTg/Prx ^-/-^* mice, there was a substantial loss of Drp2 in *ΔCPrxTg/Prx* ^-/-^ mice ( Figure 1C). This reduction in the level of Drp2 in the peripheral nerves of *ΔCPrxTg/Prx* ^-/-^ mice was reflected by a 2.6 fold reduction in the absolute value of Drp2 in *ΔCPrxTg/Prx* ^-/-^ mice by mass spectrometric analysis. Furthermore, there was a shift with age to more of the dephosphorylated lower band of Drp2, which is known to be associated with dissociation from the Prx/Drp2/Dag complex ( Figure 1C) ^15^. From previous work ^15^, we know that these changes in Drp2 levels and increased dephosphorylation should have structural consequences for the assembly and stabilization of appositions at the Schwann cell plasma membrane, and this is what we see ( Figure 1D).

**Figure 2.**
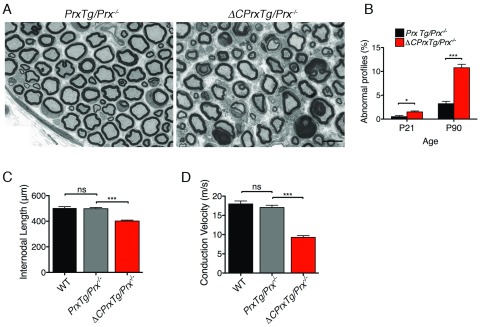
C-terminal truncation of Periaxin destabilizes myelin, and reduces internodal length and nerve conduction velocity in the PNS. ( **A**) Light microscopy of transverse sections from control ( *PrxTg/Prx ^-/-^*) and mutant ( *ΔCPrxTg/Prx ^-/-^*) quadriceps nerves at P90 show extensive abnormal profiles of myelinated axons. Size marker = 10 μm. ( **B**) There is a progressive increase in the number of abnormal profiles from P21 to P90 compared to control nerves. Mean values ± SEM, n = 3–5. ( **C**) Internodal lengths of mutant nerves are significantly reduced at P21. Mean values ± SEM, n = 4–5. ( **D**) Nerve conduction velocities of mutant nerves are significantly reduced at P21. Mean values ± SEM, n = 6–8. *P < 0.05; ***P < 0.0005; ns, not significant.

Histological analysis of mouse peripheral nerves with mutations in either the *Prx* or *Drp2* genes have previously revealed abnormal profiles of myelinated axons in transverse sections ^14, 15, 18^. This was also observed in the *ΔCPrxTg/Prx* ^-/-^ mice ( Figure 2A and B). Further, even at P21 when mutant nerves displayed a modest increase in abnormal profiles, their internodal lengths were significantly shorter ( Figure 2C). As has been argued elsewhere, this is likely to be the primary cause of the reduced nerve conduction velocities observed in mutant nerves at P21 ^14, 18^ ( Figure 2D).

## Discussion

CMT4F disease caused by C-terminal truncations of Periaxin has been difficult to explain mechanistically since the two domains that have been well-characterised as essential for the formation of the Prx/Drp2/Dag complex, namely the homodimerizing PDZ domain and the basic, Drp2 interaction domain, are both at the N-terminus of the protein ^16, 18^ ( Figure 1A). In an effort to understand why loss of the Periaxin C-terminus should cause disease we generated transgenic mice expressing either full length Periaxin or a truncated form that corresponds to the mutant protein in some patients, and we expressed these proteins in a Periaxin-null background. To our surprise, myelinating Schwann cells expressing mutated Periaxin possessed considerably less Drp2 and the protein was significantly dephosphorylated. Both factors would be expected to attenuate the formation of Prx/Drp2/Dag-rich adhesive appositions and thereby compromise the formation of Cajal bands, which, in turn, should destabilize the myelin sheath. This is what we observed, and it appeared to get worse with age.

Loss of Drp2, and the accompanying disruption of Cajal bands, has a much less severe phenotype in both mice and man than loss of Periaxin ^11, 12, 15– 17^. In mice lacking Drp2 internodal lengths are unaffected, conductions speeds are normal and demyelination is much less extensive ^15^. Therefore, Periaxin apparently has other roles in Schwann cell biology in addition to promoting the assembly of Cajal bands. The present study supports this view and identifies the C-terminus of the protein as a contributor to the regulation of Schwann cell growth, which is crucial to ensure normal conduction speeds ^14^.

## Data availability

Data are available at OSF: http://doi.org/10.17605/OSF.IO/GF56E ^24^.

Data are available under the terms of the Creative Commons Zero “No rights reserved” data waiver (CC0 1.0 Public domain dedication).

## References

[1] Rossor AM Tomaselli PJ Reilly MM: Recent advances in the genetic neuropathies. *Curr Opin Neurol.* 2016;29(5):537–548. 27584852 10.1097/WCO.0000000000000373 PMC5130159

[2] Choi YJ Hyun YS Nam SH: Novel Compound Heterozygous Nonsense *PRX* Mutations in a Korean Dejerine-Sottas Neuropathy Family. *J Clin Neurol.* 2015;11(1):92–96. 10.3988/jcn.2015.11.1.92 25628743 PMC4302186

[3] Noriega E Ramos E: New mutation in periaxin gene causing Charcot Marie Tooth disease in a Puerto Rican young male. *J Clin Neuromuscul Dis.* 2013;15(2):63–68. 10.1097/CND.0000000000000009 24263033

[4] Tokunaga S Hashiguchi A Yoshimura A: Late-onset Charcot-Marie-Tooth disease 4F caused by periaxin gene mutation. *Neurogenetics.* 2012;13(4):359–365. 10.1007/s10048-012-0338-5 22847150

[5] Nouioua S Hamadouche T Funalot B: Novel mutations in the *PRX* and the *MTMR2* genes are responsible for unusual Charcot-Marie-Tooth disease phenotypes. *Neuromuscul Disord.* 2011;21(8):543–550. 10.1016/j.nmd.2011.04.013 21741241

[6] Marchesi C Milani M Morbin M: Four novel cases of periaxin-related neuropathy and review of the literature. *Neurology.* 2010;75(20):1830–1838. 10.1212/WNL.0b013e3181fd6314 21079185

[7] Otagiri T Sugai K Kijima K: Periaxin mutation in Japanese patients with Charcot-Marie-Tooth disease. *J Hum Genet.* 2006;51(7):625–628. 10.1007/s10038-006-0408-3 16770524

[8] Kabzinska D Drac H Sherman DL: Charcot-Marie-Tooth type 4F disease caused by S399fsx410 mutation in the *PRX* gene. *Neurology.* 2006;66(5):745–747. 10.1212/01.wnl.0000201269.46071.35 16534116

[9] Parman Y Battaloglu E Baris I: Clinicopathological and genetic study of early-onset demyelinating neuropathy. *Brain.* 2004;127(Pt 11):2540–2550. 10.1093/brain/awh275 15469949

[10] Takashima H Boerkoel CF De Jonghe P: Periaxin mutations cause a broad spectrum of demyelinating neuropathies. *Ann Neurol.* 2002;51(6):709–715. 10.1002/ana.10213 12112076

[11] Boerkoel CF Takashima H Garcia CA: Charcot-Marie-Tooth disease and related neuropathies: mutation distribution and genotype-phenotype correlation. *Ann Neurol.* 2002;51(2):190–201. 10.1002/ana.10089 11835375

[12] Guilbot A Williams A Ravisé N: A mutation in periaxin is responsible for CMT4F, an autosomal recessive form of Charcot-Marie-Tooth disease. *Hum Mol Genet.* 2001;10(4):415–421. 10.1093/hmg/10.4.415 11157804

[13] Auer-Grumbach M Fischer C Papić L: Two novel mutations in the GDAP1 and PRX genes in early onset Charcot-Marie-Tooth syndrome. *Neuropediatrics.* 2008;39(1):33–38. 10.1055/s-2008-1077085 18504680 PMC3272394

[14] Court FA Sherman DL Pratt T: Restricted growth of Schwann cells lacking Cajal bands slows conduction in myelinated nerves. *Nature.* 2004;431(7005):191–195. 10.1038/nature02841 15356632

[15] Sherman DL Wu LM Grove M: Drp2 and periaxin form Cajal bands with dystroglycan but have distinct roles in Schwann cell growth. *J Neurosci.* 2012;32(27):9419–9428. 10.1523/JNEUROSCI.1220-12.2012 22764250 PMC3400949

[16] Sherman DL Fabrizi C Gillespie CS: Specific disruption of a schwann cell dystrophin-related protein complex in a demyelinating neuropathy. *Neuron.* 2001;30(3):677–687. 10.1016/S0896-6273(01)00327-0 11430802

[17] Brennan KM Bai Y Pisciotta C: Absence of *Dystrophin Related Protein-2* disrupts Cajal bands in a patient with Charcot-Marie-Tooth disease. *Neuromuscul Disord.* 2015;25(10):786–793. 10.1016/j.nmd.2015.07.001 26227883 PMC4920059

[18] Wu LM Williams A Delaney A: Increasing internodal distance in myelinated nerves accelerates nerve conduction to a flat maximum. *Curr Biol.* 2012;22(20):1957–1961. 10.1016/j.cub.2012.08.025 23022068 PMC3482659

[19] Dytrych L Sherman DL Gillespie CS: Two PDZ domain proteins encoded by the murine periaxin gene are the result of alternative intron retention and are differentially targeted in Schwann cells. *J Biol Chem.* 1998;273(10):5794–5800. 10.1074/jbc.273.10.5794 9488714

[20] Nagy E Maquat LE: A rule for termination-codon position within intron-containing genes: when nonsense affects RNA abundance. *Trends Biochem Sci.* 1998;23(6):198–199. 10.1016/S0968-0004(98)01208-0 9644970

[21] Kijima K Numakura C Shirahata E: Periaxin mutation causes early-onset but slow-progressive Charcot-Marie-Tooth disease. *J Hum Genet.* 2004;49(7):376–379. 10.1007/s10038-004-0162-3 15197604

[22] Klockenbusch C Kast J: Optimization of formaldehyde cross-linking for protein interaction analysis of non-tagged integrin beta1. *J Biomed Biotechnol.* 2010;2010: 927585. 10.1155/2010/927585 20634879 PMC2896913

[23] Gillespie CS Sherman DL Blair GE: Periaxin, a novel protein of myelinating Schwann cells with a possible role in axonal ensheathment. *Neuron.* 1994;12(3):497–508. 10.1016/0896-6273(94)90208-9 8155317

[24] Brophy PJ: 13673.WOR. *Open Science Framework.* 2018 Data Source

