# Supplementary material for: A murine model of Charcot-Marie-Tooth disease 4F reveals a role for the C-terminus of periaxin in the formation and stabilization of Cajal bands
Source: Wellcome Open Res. 2018 Mar 1;3:20. [Version 1] doi: 10.12688/wellcomeopenres.13673.1 (PMC5861512; doi:10.12688/wellcomeopenres.13673.1)
Supplement: Peer review history

# Peer review: Referee response for version 1

**DOI:** 10.21956/wellcomeopenres.14853.r31380

**Published:** 2018-03-19

**Authors:** Rudolf Martini, Dennis Klein

**Affiliations:** 1 Department of Neurology, Developmental Neurobiology, University Hospital of Würzburg, Würzburg, Germany

Periaxin, a culprit gene of CMT4F, plays important roles in the interaction of myelin and the abaxonal Schwann cell membrane by forming a Prx/Drp2/Dag complex that is necessary for the formation of cytoplasmic Schwann cell channels, called Cajal bands. As the formation of the complex is dependent on the N-terminus-related domain of Periaxin, it was unexpected that mutations affecting the C-terminus of periaxin can also cause CMT4F disease. However, the milder phenoptype of Drp2 mutants suggests an additional function of Periaxin than just acting via the formation of the N-terminus-related Prx/Drp2/Dag complex. This hypothesis was confirmed by the present study: introducing a C-terminal-deficient Periaxin transgene into the Periaxin-ko genotype led to a similarly severe phenotype as the Periaxin-ko, including vasting of Cajal bands, progredient myelin abnormalities and abnormally short internodes. The observed reduction in Drp2 was conceptionally surprising.

 The study is carefully designed, the controls are adequate (e.g., comparison of ko-mice containing full length vs truncated *Prx* transgenes) and the described data are convincing.

 As one readout is delayed conduction velocity, further studies might investigate the molecular and morphological features of the nodes. Moreover, one could address axonal loss in addition to demyelination.

 All in all, the study is a valuable additional contribution from the Brophy-group to decipher the functional roles and pathophysiology of Periaxin.

We have read this submission. We believe that we have an appropriate level of expertise to confirm that it is of an acceptable scientific standard.

# Peer review: Referee response for version 1

**DOI:** 10.21956/wellcomeopenres.14853.r31375

**Published:** 2018-03-08

**Authors:** Kleopas A. Kleopa

**Affiliations:** 1 Cyprus School of Molecular Medicine, Cyprus Institute of Neurology and Genetics (CING), Nicosia, Cyprus

CMT4F is a rare form of autosomal recessive Charcot-Marie-Tooth Disease, caused by about 24 different mutations in the *Periaxin *( *PRX*) gene that manifests with a typically painful demyelinating sensorimotor peripheral neuropathy. Periaxin forms a complex with Dystrophin-Related Protein 2 (Drp2) and Dystroglycan (Dag) at the Schwann cell membrane and this complex assembles adhesive appositions between the abaxonal surface of PNS myelin and the Schwann cell plasma membrane that are surrounded by cytoplasm-filled Cajal bands. Either the absence of Periaxin or Drp2, or the loss of the interaction between these two proteins causes disruption of the myelin sheath. The N-terminus of periaxin has been primarily shown to mediate the interaction with Drp2. However, certain CMT4F mutations result in truncation of the C-terminus suggesting that this domain of periaxin may also play a role in the stabilization of membrane appositions in myelinated fibers. The aim of the authors in this study was to clarify the mechanism by which such C-terminus mutations cause CMT4F.

 They generated a transgenic mouse line expressing a mutant periaxin with truncated C-terminus on a periaxin null background, comparing with a line expressing the full length periaxin on a null background. They demonstrate that expression of periaxin with truncation of the C-terminus results in reduced levels and progressive dephosphorylation of Drp2, associated with loss of the Drp2-positive appositions in myelinated fibers. Furthermore, morphological studies showed that C-terminal truncation of Periaxin destabilizes the myelin with progressively abnormal profiles of myelinated axons, and reduces internodal length, while nerve conduction velocity is reduced. These results lead to the conclusion that the C-terminus of periaxin mediates additional roles in Schwann cells that go beyond the direct interaction with Drp2 and the assembly of Cajal bands.

 Overall, this a well-designed, scientifically sound and clearly presented study that should lead to further investigations into the multiple functional roles of periaxin in myelinating cells and how these are impaired in CMT4F patients. The discussion could have elaborated a bit further upon these considerations and the possible underlying mechanisms.

I have read this submission. I believe that I have an appropriate level of expertise to confirm that it is of an acceptable scientific standard.
